# Colorimetry-Based Phosphate Measurement for Polymerase Elongation

**DOI:** 10.1155/2023/8296847

**Published:** 2023-01-23

**Authors:** Han Yang, Xiaoxin Ji, Xiao Li, Yunran Feng, Ke Zhang, Xuemin Guo, Zhixiong Zhong, Xin Mu

**Affiliations:** ^1^School of Pharmaceutical Science and Technology, Tianjin University, Tianjin 300072, China; ^2^Tianjin University and Health-Biotech United Group Joint Laboratory of Innovative Drug Development and Translational Medicine, Tianjin University, Tianjin 300072, China; ^3^Guangdong Provincial Key Laboratory of Precision Medicine and Clinical Translation Research of Hakka Population, Meizhou 514011, China; ^4^Meizhou People's Hospital, Meizhou 514011, China; ^5^Meizhou's Academy of Medical Sciences, Meizhou 514011, China

## Abstract

DNA detection, which includes the measurement of variants in sequences or the presence of certain genes, is widely used in research and clinical diagnosis. Both require DNA-dependent DNA polymerase-catalyzed strand extension. Currently, these techniques rely heavily on the instruments used to visualize the results. This study introduced a simple and direct colorimetric method to measure polymerase-directed elongation. First, pyrophosphate (PPi), a by-product of strand extension, is converted into phosphate (Pi). Phosphate levels were measured using either Mo-Sb or BIOMOL Green reagent. This study showed that this colorimetry can distinguish single-base variants and detect PCR products in preset stringent conditions, implicating the potential value of this strategy to detect DNA.

## 1. Introduction

Genes are encoding proteins that participate in biological activities. Sequence variants may lead to altered protein function and are sometimes pathogenic. Extensive efforts have been made to identify variants in genomes. High-throughput sequencing technology has identified many sequence variations, resulting in pathological consequences [1–4]. Targeted sequencing strategies can also be applied, such as the single-base extension (SBE) strategy. SBE normally relies on the use of dideoxynucleotides (ddNTPs), whose 2-OH is substituted by 2-H; thus, this ddNTPs cannot be extended during DNA polymerase-catalyzed strand extension. As the primer annealed to the testing template, ddNTPs were added immediately at the 3-end of the growing strand, and the reaction was stopped. Because each kind of ddNTP (ddATP, ddCTP, ddGTP, and ddTTP) carries a unique fluorescent signal, reading this signal will identify the sequence information of that single site [5–7].

Another strategy for SBE is to take advantage of the phosphorothioate (PT) bond introduced between the last 2-3 nucleotides at the 3-end of the primer. These modifications make the DNA resistant to 3-5 exonuclease cleavage from DNA polymerase but have little influence on base pairing. Mismatching at the 3-end of the primer with the DNA template results in the stall of high-fidelity DNA polymerase (e.g., Pfu) due to the inhibition of exonuclease activity and no elongation [8]. In contrast, correct annealing of primers and templates leads to strand elongation and generates double-stranded DNA (dsDNA) molecules [9, 10]. Polymerase chain reaction (PCR) products can then be visualized on agarose gels by ultraviolet (UV) irradiation [11].

In addition to detecting single-base variants, probing the presence of selected genes is also common in clinical settings, such as detecting pathogens to identify suspected individuals. Nucleic acids, antigens, and antibody-based detections are accurate and specific [12]. In establishing a detection protocol and materials, nucleic acid-based detection may be the fastest and easiest to develop. After pathogen sequencing, primers can be designed and tested immediately. Specific primers targeting pathogen nucleic acid sequences were detected using quantitative PCR (qPCR). For RNA detection, a reverse transcription reaction using random primers or oligo d(T) was performed before qPCR.

Obtaining the results of these methods relies heavily on instruments. More convenient means of detection based on colorimetry are needed and convenient in practice.

All the mentioned detections require strand elongation, and along with this process, pyrophosphate (PPi) is produced [13]. Thus, the PPi level in the reaction effectively reflects the extent of strand extension [14, 15]. Methods to detect PPi levels can start by catalyzing PPi to phosphate (Pi) by pyrophosphatase (PPase), which then reacts with maltose to form glucose 1-phosphate and glucose, which is converted by glucose oxidase to H_2_O_2_ for horseradish peroxidase (HRP) detection, for example, the use of commercial PiPer™ assay [16]. Alternatively, PPi is converted into adenosine triphosphate (ATP) by adenosine 5-phosphosulfate (APS) and ATP sulfurylase. ATP was measured using a luciferase assay with luciferin [17]. Multiple steps are required for both methods, and neither detects Pi directly.

Thereafter, we attempted to develop strategies to directly detect Pi in the absence of specific equipment for the last-step detection. Pi detection is widely used in chemical, environmental, and biological research. The Mo-Sb colorimetric method can be used for Pi detection and is mostly used with soil or other environmental samples [18–20]. BIOMOL® Green is a commercial reagent developed from Malachite Green reagent [21] and has been used successfully in the study of proteins having ATPase activity [22–24]. These two methods are simple and rapid to perform. To the best of our knowledge, neither of these two measurements for Pi has been applied to the strand elongation reaction.

In this study, we used the PPase-mediated Pi production and two colorimetric reagents (Mo-Sb colorimetry and BIOMOL® Green) for Pi detection to visualize the elongation reaction with the naked eye instead of running on the agarose gel, followed by UV detection. This study showed that these methods are reliable for detecting strand extension.

## 2. Materials and Methods

### 2.1. DNA

DNA oligonucleotides and single-stranded templates were synthesized by Sangon Biotech. pLentiCRIPSPR v2 vector (Addgene plasmid # 52961) [25] and pCMV-HA-FLAG [26] were used as the PCR templates. Genomic DNA was extracted from HeLa cells using a TIANamp Genomic DNA kit (TIANGEN, Beijing, China, Cat. No. DP304-02).

### 2.2. Native Polyacrylamide Gel Electrophoresis (PAGE) Analysis

The PAGE gel was prepared using TBE buffer, as previously reported [27]. Briefly, DNA samples after annealing and extension were analyzed on a 12% gel. Electrophoresis was performed in TBE buffer (89 mM Tris at pH 7.6, 89 mM boric acid, and 2 mM EDTA) at 115 V at room temperature (RT) and analyzed using GelRed (US Everbright, Suzhou, Jiangsu, China, Cat. No. S2001) staining.

### 2.3. Annealing and Extension

Primers and templates were mixed at the indicated concentrations and annealed in a thermal cycler. Unless otherwise mentioned, the temperature change speed followed the device's default settings. For single-cycle extension reaction, Pfu (Sangon Biotech, Shanghai, China, Cat. No. B600003-0600) and dNTP (Sangon Biotech, Shanghai, China, Cat. No. B500055-0500) were added after annealing. For extension with cycles, Pfu and dNTP were added before annealing.

### 2.4. PPase Treatment

PPase (New England Biolabs, Beijing, China, Cat. No. M2403S) was directly added to the DNA mixture after Pfu extension. After 30 min of incubation at RT, the samples were used for Pi measurements.

### 2.5. Mo-Sb Colorimetry

Mo-Sb colorimetry (Shanghai Shupei Laboratory Equipment, Shanghai, China, Cat. No. 090161) was conducted following the manufacturer's protocol. Briefly, the sample was added to the solution I provided in the kit and mixed for 30 s. Solution II, provided by the kit, was added to the mixture. The mixture was incubated at RT for 10 min. The color change was observed with the naked eye.

### 2.6. BIOMOL® Green Measurement

A phosphate standard curve was generated for every assay, and the Pi concentration was analyzed accordingly. All measurements were performed by incubating 90 *μ*l of BIOMOL® Green reagent (Enzo Life Science, Farmingdale, New York, Cat. No. BML-AK111) with 10 *μ*l of the sample at RT for 20 min. The absorbance of the resultant samples was measured quantitatively at 620 nm.

### 2.7. Streptavidin-Biotin Affinity Purification

Biotin was covalently bound to the 3-end of the adaptor sequence to enable adaptor binding to streptavidin. The reverse complementary (RV) sequences of the adaptor were synthesized at the 5-end of primers (RV-primer); thus, the adaptor was annealed to the RV-primer due to base pairing, and the unpaired part of the RV-primer could anneal to the template.

Streptavidin MagBeads (GenScript, Nanjing, China, Cat. No. L00424) was supplied as a 25% slurry in phosphate-buffered saline (PBS) at pH 7.4. For each reaction, 10 *μ*l bead slurry was used. The beads were washed three times with 100 *μ*l buffer containing 10 mM Tris at pH 7.5, and 50 mM NaCl were washed three times to remove residual ethanol. For biotin-containing DNA binding to the beads, incubation was performed at RT for 60 min on a roller. The beads were washed thrice with the same buffer mentioned above to avoid unbound DNAs. Reagents for strand extension were added directly to the beads or to the supernatants after the beads were boiled in a thermal cycler at 94°C for 5 min and pelleted.

### 2.8. PCR of Plasmid DNA and Pi Detection

In a 25 *μ*l of PCR reaction, 30 ng of plasmid DNA (pLentiCRIPSPR-v2 or pCMV-HA-FLAG) was used as a template, and 1 *μ*l of each primer (PCR FW and PCR RV) at 10 *μ*M stock was added. Pfu at a final concentration of 0.5 U/*μ*l was used to conduct strand extension. After PCR, PPase at a final concentration of 0.001 U/*μ*l was added to digest PPi, and 2 *μ*l of digested PCR product was used for BIOMOL® Green measurement. Primer sequences are shown in Table 1.

### 2.9. PCR of Genomic DNA and Pi Detection

HeLa cells were maintained in Dulbecco's Modified Eagle Medium (Meilunbio, Dalian, Liaoning, China, Cat. No. MA0212) containing 10% FBS (Cat. No.10099-141C) and 1% penicillin/streptomycin (Solarbio, Beijing, China. Cat. No. P1400). Cells were harvested for genomic DNA purification using TIANamp Genomic DNA kit (TIANGEN, Beijing, China, Cat. No. DP304-02). The genomic DNA for PCR was used at a working concentration of 2 ng/*μ*l. Primers were used at a working concentration of 10 *μ*M. The sequences of the forward and reverse primers are listed in Table 1. The annealing temperature was maintained at 69°C for 30 s. The PCR was performed for 35 cycles.

### 2.10. Statistical Analyses

**Figure 1:**
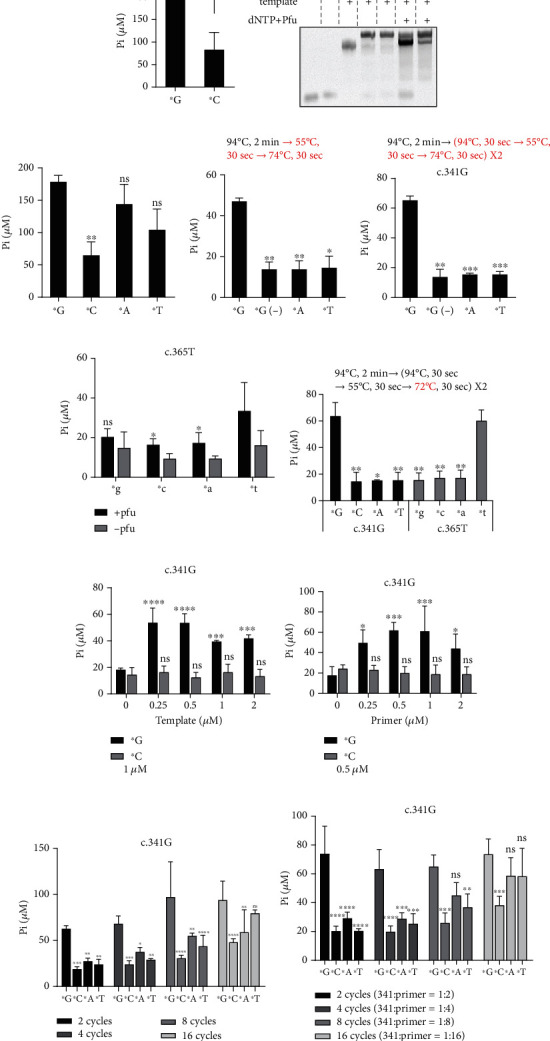
BIOMOL® Green reagent quantitatively detects single base variant. (a, b) Pi measurement using BIOMOL® Green and native PAGE analysis of samples following protocols in Figure 4(i). (c) Pi measurement using primers ended with G, C, A, or T (^∗^G, ^∗^C, ^∗^A, or ^∗^T as labeled). (d, e) Optimizing the annealing and extension conditions using primers ended with G, A, or T. (-): no Pfu in the reaction. All changed conditions are highlighted in red. (f) Pi measurement using template c.365T and its corresponding primers ended with four nucleotides, following conditions described in Figure 1(e). (g) Pi measurement using two sets of templates and primers, the revised extension condition is highlighted in red. (h) Pi measurement using 1 *μ*M of primer and different concentration of template c.341G, following conditions described in Figure 1(g). (i) Pi measurement using 0.5 *μ*M of template c.341G and different concentration of primers ended with G or C, following conditions described in Figure 1(g). (j) Pi measurement using 1 *μ*M of primer and 0.5 *μ*M of template c.341G for extension, at different cycle numbers, following conditions described in Figure 1(g). (k) Pi measurement using 1 *μ*M of primer and changed concentration of template c.341G for extension, at different cycle numbers, following conditions described in Figure 1(g). The ratio of template to primer is shown on the right. For all c.341G samples, ^∗^C, ^∗^A, and ^∗^T were calculating statistic values to ^∗^G. For all c.365T samples, ^∗^G, ^∗^C, and A were calculating statistic values to ^∗^T. Data shown are mean ± S.D., *n* = 3 independent experiments. ns: none-statistic significant, ^∗^*p* < 0.05, ^∗∗^*p* < 0.01, ^∗∗∗^*p* < 0.001, and ^∗∗∗∗^*p* < 0.0001.

**Figure 2:**
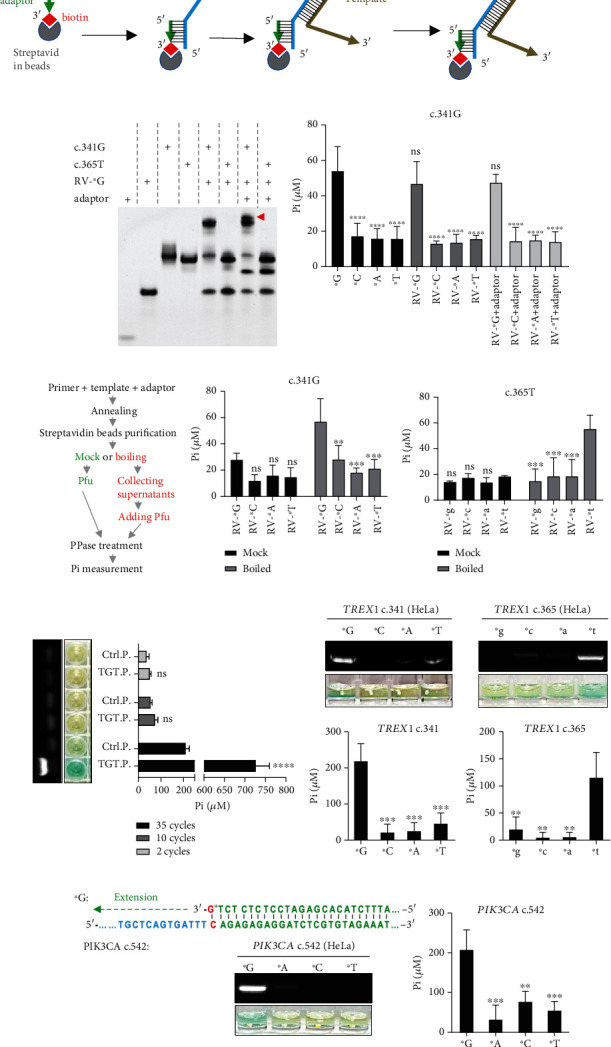
BIOMOL® Green measurement detects strand extension in streptavidin-biotin affinity purification and PCR samples. (a) Schematic diagram of the streptavidin magnetic beads binding to biotin-labeled oligos. An adaptor oligo anneals to primers (RV-primers) through its reverse complementary sequence (RV-) and binds to beads via streptavidin. The rest sequence of RV-primers selectively binds to template for strand extension. (b) Native PAGE analysis of annealing of adaptor, RV-primer, and different templates. Red arrow indicates the template: RV-primer adaptor complex. Blue arrow indicates adaptor: RV-primer duplex. (c) Tests of influence of adaptor and the RV sequence on single base extension specificity, without the use of streptavidin beads. Assays were done using conditions described in Figure 1(g). (d) Flowchart of Pi detection coupling with streptavidin-biotin affinity purification. (e, f) Purification of streptavidin beads bound to DNAs indicated are either mock treated or boiled before Pfu-driven extension. Boiled: incubate beads at 95°C for 5 min after binding. Template c.341G (e) and c.365T (f) were tested using specific primers, respectively, ended with G, C, A, or T. (g) Detection of PCR products using plasmid as template by agarose gel analysis and BIOMOL® Green measurement following PPase treatment, respectively. Ctrl.P: control plasmid; TGT.P: target plasmid. (h, i) Detection of PCR products using HeLa genomic DNA as template. The products were analyzed by both agarose gel and BIOMOL® Green measurement following PPase treatment, respectively. For all c.341G samples, ^∗^C, ^∗^A, and ^∗^T were calculating statistic values to ^∗^G. For all c.365T samples, ^∗^g, ^∗^c, and ^∗^a were calculating statistic values to ^∗^t. For all c.542 samples, ^∗^C, ^∗^A, and ^∗^T were calculating statistic values to ^∗^G. Data shown are mean ± S.D. ns: none-statistic significant. ^∗^*p* < 0.05, ^∗∗^*p* < 0.01, ^∗∗∗^*p* < 0.001, and ^∗∗∗∗^*p* < 0.0001.

Unless otherwise mentioned, the data are presented as the mean ± SD of three independent experiments. Data were analyzed using PRISM version 8.0.2. ns: none-statistic significant. ^∗^*p* < 0.05, ^∗∗^*p* < 0.01, ^∗∗∗^*p* < 0.001, and ^∗∗∗∗^*p* < 0.0001. The data set was analyzed using a *t*-test, one-way ANOVA for Figures 1(a), 1(c), 1(d), 1(e), 1(g), and 2(c), and two-way ANOVA for Figures 1(f), 1(h), 1(i), 1(j), 1(k), 2(e), 2(f), 2(g), 2(h), and 2(i).

## 3. Results

### 3.1. Single-Base Variant Detection by the Mo-Sb Colorimetry Method

**Figure 3:**
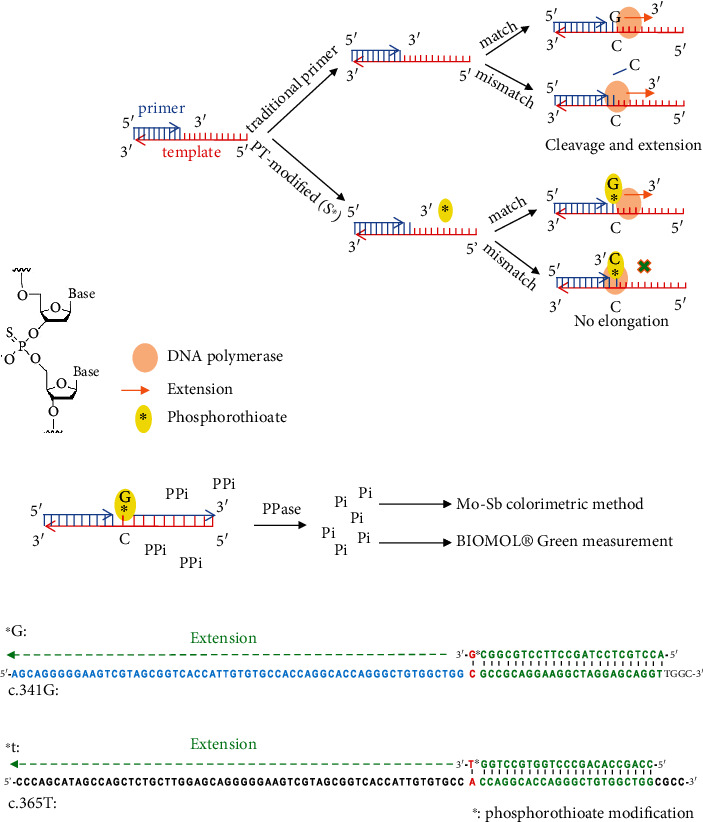
Principle of phosphorothioate-based strand extension detection. (a) Structure diagram of phosphorothioate. (b) Schematic diagram of phosphorothioate- (PT-modified-) based singe base extension. The high-fidelity DNA polymerase encodes 3 to 5 exonuclease activity and cleaves mismatching nucleotide during extension. PT-modification impedes such 3-5 exonuclease activity resulting in stalling of DNA polymerase. Red strand represents template. The shorter blue strand represents primer. Yellow circle with an asterisk inside represents PT-modification. (c) Schematic diagram of the measurement of Pi. PPase: pyrophosphatase. (d) Sequences of templates and matched primers. Template c.341G anneals to primer ^∗^G. Template c.365T anneals to primer ^∗^t. The asterisk represents PT-modification. Bases to be extended are highlighted in red.

First, we tested the SBE measurements using phosphorothioate modification. The phosphorothioate bond (Figure 3(a)) between the last two bases in oligonucleotides does not affect base pairing and elongation but inhibits the 3-5 exonuclease activity of DNA polymerase [10]. Based on this, we designed four primers that differed by the last nucleotide. The correct base pairing will trigger DNA polymerase catalyzing strand elongation; incorrect bases halt DNA polymerase because of its inability to process the cleavage of mismatched nucleotides (Figure 3(b)). During the elongation reaction, PPi was released upon base extension. It is digested by PPase to become Pi, and the resultant Pi can be measured by methods including Mo-Sb colorimetry and BIOMOL® Green (Figure 3(c)) [18–20, 22–24].

We selected two regions of the human *TREX1* gene as templates for our tests. The templates are named c.341G and c.365T, respectively, where c stands for coding sequence (CDS), and G and T represent the correct sequences at the indicated sites. These naturally occurring variants cause systemic lupus erythematosus (SLE) in humans [28, 29]. These two sites also include 3-end base pairing for both A: T and C: G. In this study, the opposite strand was used as a template sequence; therefore, the primers used the sequence of the sense strand. Examples of matched primers and templates for c.341G and c.365T are shown in Figure 3(d). The primers are named with uppercase target c.341G template and those named with lowercase target c.365T template. All sequences are shown in Table 1.

**Figure 4:**
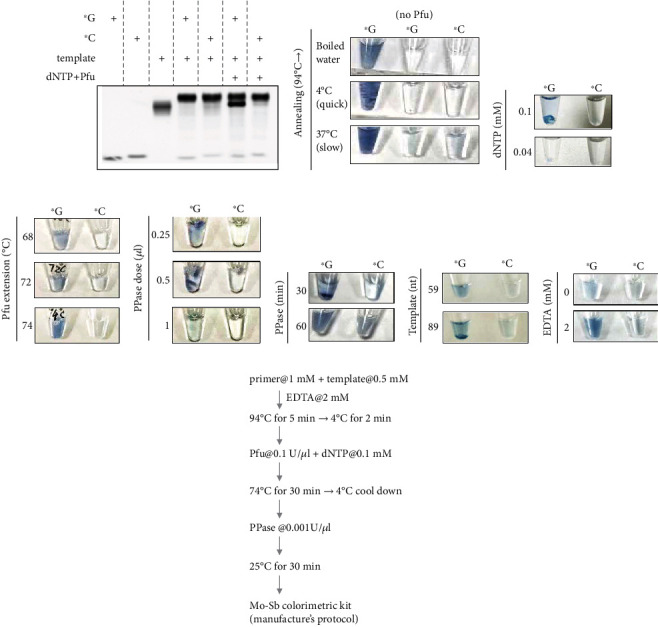
Mo-Sb colorimetry detects single base variant. (a) Native-PAGE analysis of extension on template c.341G using primers ended with G or C (^∗^G or ^∗^C as labeled). (b–h) Analysis of different assay conditions for extension using the Mo-Sb colorimetric method. (b) Annealing conditions: after heating mixed DNAs at 94°C for 5 min, they were transferred into boiled water for cooling down, thermal cycler to cooled down to 4°C using default setting or to 37°C at 0.1°C per min. (c) dNTP concentrations. (d) Pfu polymerase extension temperatures. (e) PPase concentrations. (f) PPase incubation time. (g) The length of template c.341G. (h) EDTA in annealing buffer. (i) A summarized flowchart of strand extension and Pi detection by Mo-Sb colorimetry.

We started our tests with the template c.341G and its primers ended with G or C (^∗^G or ^∗^C, where ^∗^ stands for phosphorothioate modification). Pfu DNA polymerase with 3-5 exonuclease activity was used. First, we confirmed annealing and strand extension using PAGE analysis. The primers and template were mixed and incubated at 94°C for 5 min and then transferred to boiled water for slow cooling to RT for annealing. dNTP and Pfu were added to the SBE reaction at 74°C. The samples were examined on a 12% native PAGE gel in TBE buffer. Both primers were annealed to the template to form a duplex and appeared at a higher position in the gel (Figure 4(a), lanes 4 and 5). Furthermore, Pfu-mediated chain elongation selectively produces a product with ^∗^G but not ^∗^C (Figure 4(a), lanes 6 and 7), indicating that the phosphorothioate modification coupling Pfu extension is characterized by single-base selectivity.

The Mo-Sb method detects Pi, but not PPi, in solutions [18–20]. PPi is produced during strand elongation. Treatment with PPase converted PPi into Pi, which was measured by Mo-Sb colorimetry. The reaction was optimized in terms of the following aspects: annealing conditions, dNTP concentrations, Pfu and PPase working conditions, template length for effective elongation, and EDTA. The data showed that heating the samples to 94°C for 5 min and directly cooling down to 4°C for 2 min in the thermal cycler gave a stronger signal intensity while cooling down slowly to 37°C showed comparable intensity; however, nonspecific products were weakly visualized (Figure 4(b)). 0.1 mM of dNTP is required for elongation while decreasing the concentration to 0.04 mM abolished extension reaction (Figure 4(c)). The best elongation temperature for Pfu was 74°C, compared to 68°C or 72°C (Figure 4(d)).

PPase was directly added to the samples. Interestingly, more PPase or a longer incubation time did not improve the intensity or selectivity (Figures 4(e) and 4(f)). Thereafter, we asked whether a longer template could increase color changes since the release of PPi increased. Data showed that the 89 nucleotide (nt)-long template was better than the 59 nt template (Figure 4(g)). In addition, we tested whether the presence of EDTA improved SBE detection. EDTA is a chelating reagent that binds to Mg^2+^ in a solution. Previous studies have found that the introduction of EDTA in the annealing reaction may help stabilize DNA or RNA molecules from degradation [23]. We observed that the addition of EDTA at 2 mM working concentration improved the intensity of the SBE measurements (Figure 4(h)). The tested parameters are summarized in Table 2. We summarize the procedures for SBE in the flowchart shown in Figure 4(i).

### 3.2. Single-Base Variant Detection by BIOMOL® Green Reagent

Furthermore, we investigated whether the BIOMOL® Green reagent could detect these single-base variants. Phosphate was quantified by measuring the absorbance at 620 nm. The data showed that phosphorothioate-modified primer-mediated extension distinguishes perfect match and mismatch through measurement using BIOMOL® Green (Figure 1(a)). Consistently, native PAGE analysis also confirmed an intense band appearing in ^∗^G-mediated extension (Figure 1(b), lane 6), using the optimized protocols described in Figure 4(i).

Thereafter, we included all four bases (^∗^G, ^∗^C, ^∗^A, and ^∗^T) at the 3-end of the primers for the test to determine whether the base variant could be quantitatively distinguished. The data showed that only ^∗^C selectivity failed to mediate the extension; both ^∗^A and ^∗^T gave strong Pi signals (Figure 1(c)). We reasoned that this nonspecificity may be due to the long extension time and not-tough annealing conditions. We then decreased the extension time to 30 s and changed the annealing temperature to 55°C to improve the selectivity (Figure 1(d), upper part). The data showed that this reduced the nonspecificity from ^∗^A- or ^∗^T-mediated extension (Figure 1(d)). However, it was also noted that the molar concentration of Pi released from this setting (~50 *μ*M) was lower than that released from 30 min incubation conditions (~200 *μ*M, Figure 1(c)). To potentially improve the intensity, we introduced one additional cycle for elongation (heating-annealing-extension) (Figure 1(e), upper part). The data showed that a higher Pi concentration was detected (~65 *μ*M) without increasing the nonspecific ^∗^A- or ^∗^T-mediated elongation (Figure 1(e)), although it was still much smaller than 200 *μ*M.

We then tested whether c.365T pairing with its correct primers (A: T base pairing) would result in a similar trend. Surprisingly, such assay conditions had no activity against A: T base pairing, as noted, the Pi concentrations tested were all below or near background levels (Figure 1(f)). Given that A: T base pairing has two hydrogen bonds, while C: G has three. We assumed that lowering the extension temperature from 74°C to 72°C may improve this situation. Lowering the temperature by 2°C established good conditions for A: T base pairing and C: G pairing detection (Figure 1(g)). Following this protocol, we tested the influence of the primer/template molar ratio. We observed that, at a fixed primer concentration, changes in the template concentration could have a minor influence on the extension reaction (Figure 1(h)). Again, at a fixed concentration of template, primer concentrations fluctuated slightly in the PCR reaction (Figure 1(i)). We found that further introduction of cycles to the extension did not enhance the product yield dramatically but reduced the selectivity (Figure 1(j)). Adjusting the template: primer molar ratio according to the number of cycles yielded similar results (Figure 1(k)). Thus, in our settings using BIOMOL® Green reagent, we continued to use a molar ratio of primer: template at 2 : 1 and cycles for the extension at 2. A summary of the tested parameters is presented in Table 3.

### 3.3. Biotinylated Oligos for Purification of Template and Primer-Mediated Extension

We have established an *in vitro* measuring system for single-base detection using phosphorothioate-modified primers and Pfu for the extension reaction and PPase-coupled Mo-Sb and BIOMOL® Green reagent for Pi detection. Practically, templates are always a mixture of DNA molecules, and such measurements require further purification of the correct template in the first step. Therefore, we introduced a nonhuman sequence [30] as an adaptor, and its reverse complementary sequence (RV-) was added to the 5-end of the primer (RV-primer) so that the primer could anneal to the biotinylated adaptor sequence and be purified with streptavidin-linked magnetic beads (Figure 2(a)) (Table 1 for sequence information). The beads were then washed to remove nonspecific DNA substrates before proceeding to the SBE steps.

We first tested whether adaptor and reverse complementary adaptor (RV-) sequences interfered with the extension reaction. The data showed that the RV-^∗^G primer selectively annealed to the c.341G template but not the c.365T template (Figure 2(b), lanes 5 and 6 compared to lanes 3 and 4). The adaptor was further annealed to this duplex to form a three-strand complex (Figure 2(b); lane 7, red arrow). In line with this, SBE coupled with Pi detection showed that neither the adaptor sequence nor its reverse complementary form (RV-) interfered with SBE detection (Figure 2(c)).

Thereafter, we introduced streptavidin-biotin binding procedures. A schematic flowchart is presented in Figure 2(d). We tested whether boiling the washed beads could release the bound primer and template for the SBE. As shown in Figures 2(e) and 2(f), this treatment benefited the detection of the variants.

### 3.4. Gene Detection Based on Pi Detection

Furthermore, we investigated whether our assay system could effectively measure PCR products. A pair of PCR primers (PCR FW and PCR RV, see Table 1 for sequence information) targeting the LentiCRISPR-v2 plasmid (target plasmid, TGT.P.) was used for this proof-of-concept, and the CMV-HA-FLAG plasmid (control plasmid, Ctrl.P.) was used as control. PCR was performed at different cycles, and the products were collected. The data showed that specific and strong signals could be detected at 35 cycles of PCR but not in fewer cycles, and the Pi measurement was consistent with agarose gel analysis (Figure 2(g)). Our results indicate that PPase treatment followed by BIOMOL® Green measurement is promising for detecting normal PCR to identify suspected genes or sequences.

We tested genomic DNA from HeLa cells (a human cervical cancer cell line) as a template to amplify *TREX1* sequences using the previously used c.341 and c.365 primers. Previously used phosphorothioate-modified primers were used as forward primers, and new reverse primers were synthesized to produce DNA fragments of 352 base pair (bp) and 433 bp, respectively (341 RV and 365 RV, see Table 1 for sequence information). The data showed that perfectly matched primers directed robust strand elongation, as observed by agarose gel electrophoresis. Furthermore, the BIOMOL® Green measurement effectively reflected this change (Figure 2(h)). We further tested another site in the genome belonging to the *PIK3CA* gene at 542 nt in the coding sequence, namely, PIK3CA c.542 (Figure 2(i)). Again, the agarose gel electrophoresis results were consistent with the colorimetric measurements (Figure 2(i)).

## 4. Discussion

Studies in inorganic chemistry, organic chemistry, and molecular biology have highlighted each other. One example is the combination of nanomaterials [31–33] and biological components in disease treatment [34]. In this study, we report strategies using phosphate-detecting reagents for biological measurements, detection of both single-base variants using phosphorothioate primers, and PCR amplification with normal primers via colorimetric methods. Under ideal conditions, where only the primer and template were present, this method gave an intense and specific detection of single-base variants (Figures 4 and 1). Bead purification reduced sensitivity or intensity, but still gave more than 2-fold change compared to the correct base pairing to the incorrect base pairing (Figures 2(e) and 2(f)). Moreover, we applied this strategy to measure the common PCR products to detect the presence of a specific sequence (Figures 2(g)–2(i)). In summary, we conclude that the colorimetric phosphate measurements described here are effective for strand extension detection.

This method relies on the production of Pi, which is obtained from PPi. Both forms of phosphate are important materials in the field of chemistry [35, 36]. PPi is released after DNA polymerase-mediated strand elongation [37, 38]. Several methods have been previously applied to detect PPi, either by converting it into ATP for further measurement [22–24] or by digesting it to Pi for detection [18–20]. In our setting, we found that there was no need to change the buffer or filter out existing proteins (such as DNA polymerase) for PPase treatment, and the detection of Pi by both Mo-Sb colorimetry and BIOMOL® Green reagent did not require additional treatment of the existing proteins. In our settings, Mo-Sb colorimetry provided quick results and did not require additional equipment for examination. However, it sometimes showed fluctuations among assays, as shown in Figure 4. BIOMOL® Green measurements can be performed in 20 min and detected by either a microplate reader or the naked eye. The exact molar concentration of phosphate can be determined using a standard phosphate solution. We observed that streptavidin-biotin affinity purification reduced the sensitivity and specificity of the reaction. This may be due to the loss of beads during the mixing and washing steps, and streptavidin covalently linked to the solid phase for purification may be helpful.

In addition, the success of measuring PCR products using BIOMOL® Green reagent (Figures 2(g)–2(i)) makes this protocol a promising tool for detecting the presence of suspected or mutated genes without the need for qPCR, for example, in the case of detecting pathogens in samples. We noticed that nonspecific amplification using phosphorothioate-modified primers appeared in some tests (Figures 2(h) and 2(i)), suggesting that the mismatched nucleotide was cleaved by the 3-5 exonuclease activity of Pfu, followed by strand elongation continued to go. It has been reported that five tandem bonds would thoroughly block exonuclease activity [39], which would explain our observations.

In summary, the use of Mo-Sb or BIOMOL® Green reagents to measure phosphate levels provides inexpensive, quick, and reliable methods that can potentially be applied in gene detection or genetic variance tests. However, this method does not allow the intrinsic identification of specific nucleotides but relies on preset stringent conditions to predict the outcome and nature of the added nucleotides. This strategy is based on the production of PPi; thus, nonspecific strand extension will disturb the measurement and give false-positive conclusions. Moreover, the results cannot assess the size of the amplified products; thus, agarose gel electrophoresis is needed to clarify this. Appropriate primer design and amplification condition optimization are required as the first step. The refinement of this method to overcome these limitations is a direction for future work.

## Figures and Tables

**Table 1:** DNA sequence information. All DNA sequences described are listed below.

DNA sequences (5-3)	
Primer ^∗^G	Acctgctcctagccttcctgcggc^∗^g
Primer ^∗^C	Acctgctcctagccttcctgcggc^∗^c
Primer ^∗^A	Acctgctcctagccttcctgcggc^∗^a
Primer ^∗^T	Acctgctcctagccttcctgcggc^∗^t
c.341G (59 nt)	Tgtgccaccaggcaccagggctgtggctggcgccgcaggaaggctaggagcaggttggc
c.341G (89 nt)	agcagggggaagtcgtagcggtcaccattgtgtgccaccaggcaccagggctgtggctggcgccgcaggaaggctaggagcaggttggc
Primer ^∗^g	Ccagccacagccctggtgcctgg^∗^g
Primer ^∗^c	Ccagccacagccctggtgcctgg^∗^c
Primer ^∗^a	Ccagccacagccctggtgcctgg^∗^a
Primer ^∗^t	Ccagccacagccctggtgcctgg^∗^t
c.365T	cccagcatagccagctctgcttggagcagggggaagtcgtagcggtcaccattgtgtgccaccaggcaccagggctgtggctggcgcc
Adaptor-biotin	Aatgatacggcgaccaccgagatct-biotin
RV-primer ^∗^G	Agatctcggtggtcgccgtatcattacctgctcctagccttcctgcggc^∗^g
RV-primer ^∗^C	Agatctcggtggtcgccgtatcattacctgctcctagccttcctgcggc^∗^c
RV-primer ^∗^A	Agatctcggtggtcgccgtatcattacctgctcctagccttcctgcggc^∗^a
RV-primer ^∗^T	Agatctcggtggtcgccgtatcattacctgctcctagccttcctgcggc^∗^t
RV-primer ^∗^g	Agatctcggtggtcgccgtatcattccagccacagccctggtgcctgg^∗^g
RV-primer ^∗^c	Agatctcggtggtcgccgtatcattccagccacagccctggtgcctgg^∗^c
RV-primer ^∗^a	Agatctcggtggtcgccgtatcattccagccacagccctggtgcctgg^∗^a
RV-primer ^∗^t	Agatctcggtggtcgccgtatcattccagccacagccctggtgcctgg^∗^t
PCR FW	aatgatacggcgaccaccgagatctacactctttccctacacgacgctcttccgatcttaagtagaggctttatatatcttgtggaaaggacgaaacacc
PCR RV	caagcagaagacggcatacgagattcgccttggtgactggagttcagacgtgtgctcttccgatctccgactcggtgccactttttcaa
341 RV	Gcgtgagcatccacccaccgcagca
365 RV	Cctggttgtggccaggtgtgcagtggt
PIK3CA FW ^∗^G	Agctcaaagcaatttctacacgagatcctctctct^∗^g
PIK3CA FW ^∗^C	Agctcaaagcaatttctacacgagatcctctctct^∗^c
PIK3CA FW ^∗^A	Agctcaaagcaatttctacacgagatcctctctct^∗^a
PIK3CA FW ^∗^T	Agctcaaagcaatttctacacgagatcctctctct^∗^t
PIK3CA RV	Gcatttaatgtgccaactaccaatgtagtatgattttccac

^∗^: phosphorothioate bond.

**Table 2:** Comparison of conditions tested in Figure 4.

		Strengthen	Nonspecificity	Conclusion
Annealing	Boiled water	++		
	**4**°C (*quick*)	**+++**		*√*
	37°C (slow)	+++	+	

Extension	68°C	+		
	72°C	++		
	**74**°C	**+++**		*√*

PPase dose	0.25 *μ*l	+		
	**0.50** *μ*l	**++**		*√*

PPase time	1.00 *μ*l	—		
	**30** min	**++**		*√*
	60 min	+		

Template	59 nt	+		
	**89** nt	**++**		*√*

EDTA	0 mM	+		
	**2** mM	**++**		*√*

The conditions used for the next assay are shown on the right lane and highlighted in boldface.

**Table 3:** Comparison of conditions tested in Figure 1.

		Strengthen	Nonspecificity	Conclusion
Annealing and cycles	55°C, 1 cycle	++		
	**55°C, 2** cycle	**+++**		**√**

Template	0.25 *μ*M	+++		
	**0.50** *μ*M	**+++**		**√**
	1.00 *μ*M	++		
	2.00 *μ*M	++		

Primer	0.25 *μ*M	++		
	0.50 *μ*M	+++		
	**1.00** *μ*M	**+++**		**√**
	2.00 *μ*M	++		

Cycles	**2**	**+++**		**√**
	4	+++		
	8	+++	+	
	16	+++	++	

The conditions used for the next assay are shown on the right lane and highlighted in boldface.

## Data Availability

Source data can be obtained upon reasonable request to the corresponding authors.

## Abbreviation

PPi: Pyrophosphate
Pi: Phosphate
SBE: Single-base extension
ddNTP: Dideoxynucleotide
PT: Phosphorothioate
dsDNA: Double-stranded DNA
PCR: Polymerase chain reaction
UV: Ultraviolet
qPCR: Quantitative PCR
PPase: Pyrophosphatase
HRP: Horseradish peroxidase
ATP: Adenosine triphosphate
APS: Adenosine 5′-phosphosulfate
RT: Room temperature
PBS: Phosphate-buffered saline
CDS: Coding sequence
SLE: Systemic lupus erythematosus
nt: Nucleotide
bp: Base pair.
